# Unpeeling the onion: Digital triage and monitoring of general practice, private psychiatry, and psychology

**DOI:** 10.1177/10398562231222826

**Published:** 2023-12-19

**Authors:** Stephen Allison, Tarun Bastiampillai, Stephen Kisely, Jeffrey CL Looi

**Affiliations:** College of Medicine and Public Health, 1065 Flinders University, Adelaide, SA, Australia; and Consortium of Australian-Academic Psychiatrists for Independent Policy and Research Analysis (CAPIPRA), Canberra, ACT, Australia; College of Medicine and Public Health, 1065 Flinders University, Adelaide, SA, Australia; Consortium of Australian-Academic Psychiatrists for Independent Policy and Research Analysis (CAPIPRA), Canberra, ACT, Australia; and Department of Psychiatry, 2541 Monash University, Clayton, VIC, Australia; Consortium of Australian-Academic Psychiatrists for Independent Policy and Research Analysis (CAPIPRA), Canberra, ACT, Australia; School of Medicine, Princess Alexandra Hospital, 1974 The University of Queensland, Woolloongabba, QLD, Australia; and Departments of Psychiatry, Community Health and Epidemiology, Dalhousie University, Halifax, NS, Canada; Consortium of Australian-Academic Psychiatrists for Independent Policy and Research Analysis (CAPIPRA), Canberra, ACT, Australia; and Academic Unit of Psychiatry and Addiction Medicine, School of Medicine and Psychology, Canberra Hospital, 2219 The Australian National University, Canberra, ACT, Australia

**Keywords:** digital triage, modelling and simulation, mental health policy

## Abstract

**Objective:**

The Australian federal government is considering a ‘digital front door’ to mental healthcare. The Brain and Mind Centre at the University of Sydney has published a discussion paper advocating that the government should adopt a comprehensive model of digital triage and monitoring (DTM) based on a government-funded initiative Project Synergy ($30 million). We critically examine the final report on Project Synergy, which is now available under a Freedom of Information request.

**Conclusion:**

The DTM model is disruptive. Non-government organisations would replace general practitioners as care coordinators. Patients, private psychiatrists, and psychologists would be subjected to additional layers of administration, assessment, and digital compliance, which may decrease efficiency, and lengthen the duration of untreated illness. Only one patient was deemed eligible for DTM, however, during the 8-month regional trial of Project Synergy (recruitment rate = 1/500,000 across the region). Instead of an unproven DTM model, the proposed ‘digital front door’ to Australian mental healthcare should emphasise technology-enabled shared care (general practitioners and mental health professionals) for the treatment of moderate-to-severe illness.

The Australian federal government Mental Health Reform Advisory Committee is considering a ‘digital front door’ to mental health services.^1^ The Brain and Mind Centre at University of Sydney has published a policy discussion paper advocating that the government should adopt a comprehensive model of digital triage and monitoring (DTM), based on an Australian Government Department of Health-funded initiative Project Synergy ($30 million) delivered by InnoWell Pty Ltd – a joint venture between the University of Sydney and PwC(Australia).^2^

The main premise of the DTM model is that ‘*New digital technologies can drive more effective and accurate assessment, tracking and calibration of individual consumer needs*’ (p. 15)^2^ than the existing system of general practitioner (GP) shared care with private psychiatrists, psychologists, and other mental health professionals.^3^ We critically examine the evidence that digital technologies are more effective than GPs as care coordinators and that patient access to mental healthcare is improved.

## The digital triage model

**Figure 1.**
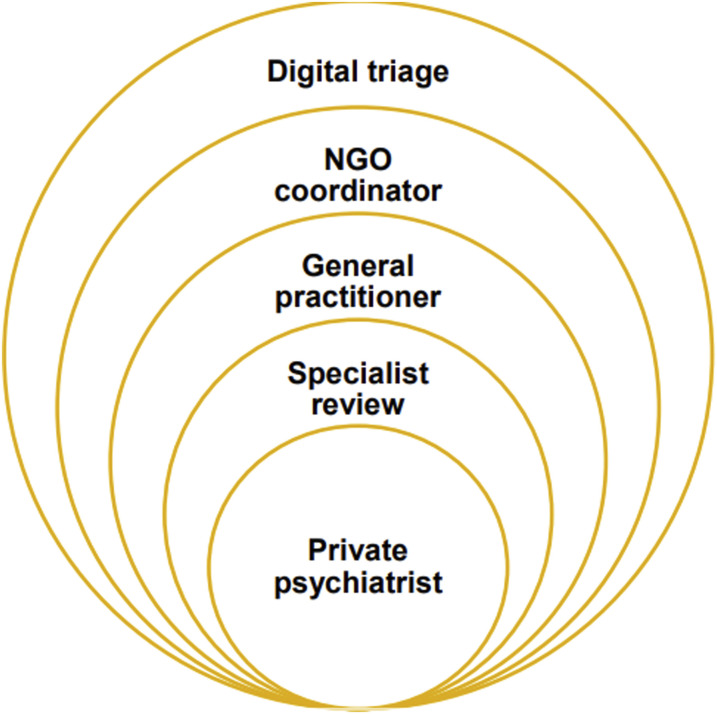
Onion-like new layers of regional mental health care.

As shown in Figure 1, the main elements of the proposed DTM model are as follows:

1. A newly funded national digital infrastructure for triage and monitoring, based on the Project Synergy digital platform,
2. Primary Health Networks (PHNs) commissioning Non-Government Organisations (NGOs) as regional coordinators of technology-enabled patient triage and tracking, and
3. A new ‘specialised assessment and review’ function for Medicare-subsidised private psychiatrists and psychologists.^2,4,5^

Under the DTM model, patients would initially access a federally funded digital platform and complete a series of routine self-report questionnaires.^4^ Algorithms would then triage patients to the following levels of care based on severity: self-management (e.g. apps and e-tools), ambulatory care (e.g. GPs and psychiatrists/psychologists), or acute services (e.g. public hospital emergency departments).^4^

In the DTM model, PHNs would commission NGOs to provide regional coordination services to oversee digital triage (Figure 1). *These technology-enabled NGO services would displace GPs from their central roles as patient advocates including relational support and care coordination*.^3^ It is probable that these additional services would require considerable expansion of federal funding for PHNs and NGOs.^3^ Surprisingly for such a large-scale program (technology plus services), the DTM model is un-costed.^2^

Roles for private psychiatrists and psychologists are unclear in the DTM model but include a new specialised assessment and review function. This seems to be an expanded role beyond Medicare fee-for-service treatment, but insufficient detail is provided to fully understand this function.^2^ For example, it is suggested that PHNs/NGOs would be able to directly access psychiatrists, but it not clear how this would happen unless PHNs employed psychiatrists or contracted their services.^2^

The regional DTM model adds additional layers of regional administration (by PHNs and NGOs), digital compliance (patients and health professionals adding information on a digital platform), and assessment (the new specialist assessment and review function) to primary and secondary private mental health care, similar to the structure of an onion (Figure 1), which is likely to decrease overall efficiency.^2^ This is unfortunate, as one of the relative strengths of the Australian health system is reasonably good administrative efficiency.^6^ Australian patients and healthcare professionals spend less time completing online forms, paperwork, and other administrative tasks than in most other high-income countries, especially the USA.^6^

Most importantly, there are additional concerns with the DTM model for patients with moderate-to-severe-mental illness who may face longer and more complex journeys to care, potentially delaying treatment from a private psychiatrist, and thereby lengthening the duration of untreated illness (Figure 1).^2^ Patients with lower digital literacy and reduced access to the technology may experience additional barriers if care is predicated on digital triage. Shared care between GPs and psychiatrists may be disrupted, since treatment decisions would be made at other levels of the system.

## The regional trial of digital triage

The DTM model suggests that any losses in efficiency and delays in treatment would be offset by better outcomes from digital coordination.^7^ For instance, introducing digital triage in North Coast region of New South Wales (NSW) (approximately 500,000 people) is predicted to reduce mental health-related presentations to regional hospitals by 10%, lower self-harm hospitalisations and regional suicide mortality by 6%, and reduce the community prevalence of anxiety and depression by 3%.^7^

Based on these promising findings, Project Synergy conducted a regional trial of digital pre-clinic triage in North Coast NSW from May to December 2020, during the COVID-19 pandemic.^5^ The regional North Coast NGO community team redesigned their service model around the Project Synergy digital platform.^5^ The trial aimed to assess outcomes for patients, health professionals, and regional services.^4,8^

The final report on Project Synergy, available under a Freedom of Information request, indicates that *only one patient was deemed eligible by the regional NGO to use the Project Synergy digital platform during the 8-month trial*.^5^ Thus, the recruitment rate was approximately 1/500,000 in North Coast NSW, so there was no opportunity to assess the outcomes of DTM for patients, health professionals, and regional services. In particular, DTM did not demonstrate superiority to GP care coordination. Despite these limited real-world outcomes, the technology-enhanced pathway of care developed in the North Coast trial forms the basis for the DTM model.^2–4,8,9^

In the absence of substantive evidence of better real-world outcomes, the DTM model appears to be reliant on theoretical modelling. There is good reason, however, to doubt the accuracy of this kind of modelling, which forecasts substantial increases in population distress and suicide in Australia during the COVID-19 pandemic that did not eventuate.^10,11^ The modelling for North Coast NSW predicted a public mental health crisis with a 23% increase in suicide mortality during the pandemic.^7^ In fact, suicide rates were either flat or fell in the State of NSW during the pandemic.^11^

Given these inaccurate predictions, Glozier and colleagues suggest: ‘*We need an honest evaluation of the assumptions and performance of the models currently used to inform policy*’ (p. 14).^11^ This suggestion could be extended to a careful evaluation of the evidence for digital triage. Thus far, Project Synergy has not provided substantive evidence that digital technologies improve the accessibility, effectiveness, and/or efficiency of primary and secondary mental healthcare.^12^

## Prioritising the therapeutic alliance

The DTM model is consistent with trends in both government and commerce to reduce costs by shifting consumer-facing services online and reducing in-person services. However, digital services have limitations, and it can be challenging to solve complex problems online. Consumers of online businesses and government agencies can become stuck in recurring digital service loops that do not lead to a solution.

In an analogous manner, patients living with moderate-to-severe mental illness might find it difficult to navigate the web-based resources of the DTM model.^2,12^ We can envision a scenario where a new patient in the midst of an acute suicidal crisis, goes online, completes a series of self-report measures, and is prompted to seek emergency help – this is unlikely to be a sufficiently helpful process.^4^

Complex mental health problems are not readily addressed by automated systems. Patients need trusting relationships with their GP, private psychiatrist, and/or psychologist. The therapeutic alliance is fundamental to effective mental healthcare and is not readily supplanted by apps and online advice.

In conclusion, there is no substantive evidence from Project Synergy to date that DTM ensures patients are ‘*on the right track from the start’*.^2,12^ Without this evidence, the proposed ‘digital front door’ should support rather than disrupt the GP’s roles as patient advocates and care coordinators. Also, patients and private psychiatrists/psychologists should not be subjected to additional layers of administration, assessment, and digital compliance without convincing evidence for improved access to specialist care and better clinical outcomes. The proposed ‘digital front door’ should build on the relative efficiency of the Australian health system by supporting patient access to in-person shared care from their chosen professionals for the treatment of moderate-to-severe illness.
